# The S. D. Gross Professorship of Pathological Anatomy

**Published:** 1884-09

**Authors:** D. Hayes Agnew, J. M. Barton

**Affiliations:** Chairman; 1344 Spruce Street, Phila.; Secretary, 1344 Spruce Street, Phila.


### CORRESPONDENCE.

---

THE S. D. GROSS PROFESSORSHIP OF PATHOLOGICAL ANATOMY.

American surgery has had no better exponent than Samuel D. Gross; none so honored abroad and at home by institutes of learning; none more revered by his associates and his pupils. His long and brilliant professional career deserves the perpetuation of his name in close association with medical tuition.

In furtherance of this object, the Alumni Association of Jefferson Medical College has inaugurated a movement to secure, in some medical school, the endowment of a Memorial Professorship, to be designated The S. D. Gross Professorship of Pathological Anatomy.

The profession at large, the personal friends of the late Professor Gross, and others interested in elevating the standard of medical education, are cordially invited by the undersigned to participate in this graceful recognition of conduct and services which have largely helped to establish the high standard of excellence to which surgery has attained throughout the United States, and served so much to dignify the repute of American medicine.

Contributions may be sent to Dr. R. J. Dunglison, Treasurer, Lock Box 1274, Philadelphia P. O., and will be acknowledged in the columns of the *Medical News* of Philadelphia.

D. HAYES AGNEW, M. D., *Chairman.*
J. M. BARTON, M. D., *Secretary,*
1344 *Spruce Street, Phila.*

COMMITTEE.

Dr. D. Hayes Agnew, Dr. Samuel Ashhurst, Dr. Wm. B. Atkins, Dr. Roberts Bartholow, Dr. J. M. Barton, Dr. J. Solis Cohen, Dr. J. DaCosta, Dr. R. J. Dunglison, Dr. N. L. Hatfield, Dr. I. M. Hays, Dr. P. J. Horwitz, Dr. Wm. Hunt, Dr. Joseph Leidy, Dr. R. J. Levis, Dr. J. Ewing Mears, Dr. S. Weir Mitchell, Dr. Geo. R. Morehouse, Dr. Milton B. Musser, Dr. Andrew Nebinger, Dr. Theo. Parvin, Dr. Wm. H. Parish, Dr. William Pepper, Dr. W. S. W. Ruschenberger, Dr. Henry H. Smith, Dr. Alfred Stillé, Dr. Wm. Thomson, Dr. Laurence Turnbull, Dr. Wm. H. Warder, Dr. J. C. Wilson.

AUXILIARY COMMITTEE.

*Alabama.*—Dr. W. O. Baldwin, Montgomery; Dr. C. H. Mastin, Mobile.

*Arkansas.*—Dr. J. M. Keller, Hot Springs; Dr. P. O. Hooper, Little Rock.

*California.*—Dr. James. F. Foulkes, Oakland; Dr. G. A. Shurtleff, Sacramento; Dr. R. B. Cole, San Francisco.

*Canada.*—Dr. LeBaron Botsford, Halifax, N. S.; Dr. J. P. Steves, St. Johns, N. B.; Dr. W. T. Aikens, Toronto.

*Colorado.*—Dr. Charles Denison, Denver.

*Connecticut.*—Dr. B. F. Silliman, New Haven; Dr. A. M. Shew, Middletown; Dr. C. W. Chamberlain, Hartford; Dr. G. L. Porter, Bridgeport.

*Dakota.*—Dr. Hector Galloway, Fargo.

*Delaware.*—Dr. L. P. Bush, Wilmington; Dr. W. Marshall, Milford.

*District of Columbia.*—Dr. J. S. Billings, Washington; Dr. J. M. Toner, Washington.

*Florida.*—Dr. E. T. Sabal, Jacksonville.

*Georgia.*—Dr. W. F. Westmoreland, Atlanta; Dr. A. W.

Calhoun, Atlanta; Dr. H. F. Campbell, Augusta; Dr. Robert Battey, Rome; Dr. A. J. Semmes, Savannah.

*Illinois.*—Dr. Moses Gunn, Chicago; Dr. N. S. Davis, Chicago; Dr. David Prince, Jacksonville; Dr. F. B. Haller, Vandalia; Dr. J. H. Rauch, Springfield; Dr. W. A. Byrd, Quincy; Dr. H. T. Coffey, Peoria.

*Indiana.*—D. J. A. Comingor, Indianapolis; Dr. E. S. Elder, Indianapolis; Dr. J. R. Weist, Richmond; Dr. J. E. Link, Terre Haute.

*Iowa.*—Dr. F. Staples, Dubuque; Dr. J. C. Hughes, Keokuk; Dr. W. F. Peck, Davenport; Dr. W. S. Robertson, Muscatine; Dr. A. G. Field, Des Moines.

*Kansas.*—Dr. D. J. Holland, Atchison.

*Kentucky.*—Dr. S. James, Frankfort; Dr. J. W. Thompson, Paducah; Dr. D. W. Yandell, Louisville; Dr. J. S. Bruce, Lexington; Dr. L. Beecher Todd, Lexington.

*Louisiana.*—Dr. T. G. Richardson, New Orleans.

*Maine.*—Dr. Alonzo Garcelon, Lewiston; Dr. D. C. Smith, Portland.

*Maryland.*—Dr. Christopher Johnston, Baltimore; Dr. Allen P. Smith, Baltimore; Dr. J. H. Jamar, Elkton; Dr. W. H. McCormick, Cumberland.

*Massachusetts.*—Dr. R. L. Hodgdon, Arlington; Dr. D. W. Cheever, Dr. J. Collins Warren, and Dr. E. Gay, Boston; Dr. Nathan Allen, Lowell; Dr. J. H. Mackie, New Bedford.

*Michigan.*—Dr. G. K. Johnson, Grand Rapids; Dr. T. A. McGraw, Detroit; Dr. W. Brodie, Detroit.

*Minnesota.*—Dr. A. J. Stone, St. Paul; Dr. R. J. Hill, Minneapolis.

*Missouri.*—Dr. E. H. Gregory, St. Louis; Dr. A. P. Lankford, Kansas City.

*Mississippi.*—Dr. A. G. Smythe, Baldwin; Dr. Wirt Johnson, Jackson.

*Nebraska.*—Dr. S. D. Mercer, Omaha.

*New Hampshire.*—Dr. I. W. Parsons, Portsmouth; Dr. J. L. Sweet, Newport.

*New Jersey.*—Dr. B. A. Watson, Jersey City; Dr. Abraham Coles, Newark; Dr. T. J. Marsh, Paterson; Dr. William Elmer, Jr., Trenton; Dr. E. L. B. Godfrey, Camden; Dr. Jas. S. Green, Elizabeth.

*New York.*—Dr. A. Van Derveer, Albany; Dr. J. C. Hutchinson, Brooklyn; Dr. W. S. Tremaine, Buffalo; Dr. T. H. Squire, Elmira; Dr. F. S. Dennis, Dr. L. A. Sayre, Dr. Louis Elsberg, and Dr. Austin Flint, New York; Dr. E. M. Moore, Rochester; Dr. R. B. Bontecou, Troy; Dr. E. N. Brush, Utica; Dr. H. D. Didama, Syracuse.

*North Carolina.*—Dr. Eugene Grissom, Raleigh; Dr. R. L. Payne, Lexington.

*Ohio.*—Dr. P. S. Conner, Cincinnati; Dr. Starling Loving, Columbus; Dr. X. C. Scott, Cleveland; Dr. J. T. Reeve, Dayton; Dr. G. D. Colamer, Toledo; Dr. Alex. Dunlap, Springfield.

*Oregon.*—Dr. H. Carpenter, Portland.

*Pennsylvania.*—Dr. Cyrus B. King, Allegheny; Dr. E. G. Martin, Allentown; Dr. W. S. Ross, Altoona; Dr. E. P. Allen, Athens; Dr. P. B. Breinig, Bethlehem; Dr. Jos. Foulke, Buckingham; Dr. A. M. Miller, Bird-in-Hand; Dr. J. L. Suesserott, Chambersburg; Dr. H. B. Van Valzah, Clearfield; Dr. W. B. Ulrich, Chester; Dr. J. K. Lineaweaver, Columbia; Dr. F. Swartzlander, Doylestown; Dr. Traill Green, Easton; Dr. J. L. Stewart, Erie; Dr. R. L. McCurdy, Freeport; Dr. J. W. C. O'Neal, Gettysburg; Dr. T. J. Dunott, Harrisburg; Dr. W. B. Lowman, Johnstown; Dr. M. L. Herr, Lancaster; Dr. S.

T. Davis, Lancaster; Dr. R. Leonard, Mauch Chunk; Dr. T. C. Stellwagen, Media; Dr. R. Rothrock; McVeytown; Dr. Ellis Phillips, New Haven; Dr. A. M. Pollock, Pittsburgh; Dr. Hiram Corson, Plymouth Meeting; Dr. W. Murray Weidman, Reading; Dr. H. Isaac Jones, Scranton; Dr. A. P. Brubaker, Somerset; Dr. G. F. Horton, Terrytown; Dr. G. O. Moody, Titusville; Dr. Varian, Titusville; Dr. D. V. Shoemaker, Warren; Dr. Isaac Massey, West Chester; Dr. R. Davis, Wilkes Barre; Dr. Thos. H. Helsby, Williamsport; Dr. J. W. Kerr, York.

*Rhode Island.*—Dr. E. T. Caswell, Providence.

*South Carolina.*—Dr. R. A. Kinloch, Charleston.

*Tennessee.*—Dr. Richard Douglas, Nashville; Dr. W. T. Briggs, Nashville; Dr. B. B. Lenoir, Lenoirs.

*Texas.*—Dr. H. C. Ghent, Belton; Dr. T. J. Heard, Galveston; Dr. D. R. Wallace, Waco.

*Utah.*—Dr. S. O. L. Potter, Salt Lake City.

*Vermont.*—Dr. Joseph Draper, Brattleboro.

*Virginia.*—Dr. Hunter McGuire, Richmond; Dr. T. J. Cullen, Petersburg; Dr. J. L. Cabell, University of Virginia.

*West Virginia.*—Dr. J. E. Reeves, Wheeling.

*Wisconsin*—Dr. T. P. Russell, Oshkosh; Dr. N. Senn, Milwaukee; Dr. J. T. Reeve, Appleton.

*Foreign.*—Dr. Th. Billroth, Vienna, Austria; Sir James Paget, London, England; Dr. Rudolph Virchow, Berlin, Prussia; Mr. Thomas Aannandale, Edinburgh, Scotland; Dr. Macleod, Glasgow, Scotland; Mr. Jolliffe Tufnel, Dublin, Ireland; Mr. Reginald Harrison, Liverpool, England.

